# Sequential exposure to specific antibody escape mutations may program neutralization breadth during subtype A HIV-1 infection

**DOI:** 10.1186/1742-4690-9-S2-P104

**Published:** 2012-09-13

**Authors:** MK Murphy, L Yue, R Pan, S Boliar, A Sethi, E Karita, SA Allen, E Cormier, JE Robinson, S Gnanakaran, E Hunter, X Kong, CA Derdeyn

**Affiliations:** 1 Emory University, Atlanta, GA, USA; 2 New York University School of Medicine, New York, NY, USA; 3 Los Alamos National Laboratory, Los Alamos, NM, USA; 4 Projet San Francisco, Kigali, Rwanda; 5 International AIDS Vaccine Initiative, London, UK; 6 Tulane University School of Medicine, New Orleans, LA, USA

## Background

Mechanisms that expand the otherwise narrow neutralization capacity observed during early HIV-1 infection are currently undefined, but multiple lines of evidence suggest that the ability to elicit broad and potent neutralizing antibodies (nAbs) via vaccination could increase the protective efficacy of immunization.

## Methods

Here we characterized the initial nAb response in a subtype A HIV-1-infected Rwandan seroconverter and investigated how consequent immune events influenced the downstream development of cross-clade breadth. Autologous envelope (Env) glycoproteins from the transmitted/founder virus and twenty longitudinal nAb escape variants were utilized to define the neutralization targets of autologous plasma and monoclonal antibodies (mAbs), the latter of which were also examined genetically and structurally through crystallization. Heterologous Env glycoproteins from nine cross-clade variants were used to determine neutralization breadth.

## Results

Initially, nAbs targeted a single region of gp120 at the base of V3 involving the alpha2 helix. A single amino acid change at one of three positions conferred early escape from plasma nAbs. Then, two autologous mAbs, revealed to have flat epitope contact surfaces, typified the second wave of nAb pressure and neutralized escape Envs carrying the defined V3/alpha2 helix substitutions in a manner dependent on immunoglobulin light chain variable domain modifications. Subsequent mAb resistance arose in later Envs through alteration of two glycan motifs previously implicated in the development of nAb breadth. Finally, three-year autologous plasma displayed moderate neutralization breadth and most potently neutralized heterologous Envs containing the altered glycan motifs.

## Conclusion

Our data demonstrate that the V3-proximal nAb epitope originally recognized in this individual elicited strain-specific mAbs and that glycan-mediated escape from these mAbs likely initiated the development of heterologous neutralization breadth. These findings suggest that epitope localization and the resultant routes of viral immune evasion, which include exposure to a specific sequence of nAb escape variants, drive humoral immune responses toward cross-clade viral recognition.

